# Effect of polycaprolactone nanofibers loaded with oxytetracycline hydrochloride and zinc oxide as an adjunct to SRP on GCF lipocalin-2 levels in periodontitis patients: A clinical and laboratory study

**DOI:** 10.34172/japid.2022.024

**Published:** 2022-11-20

**Authors:** Mohamad Fathi Mohamad Taher Alkayali, Farid A. Badria, Azza Abdel Baky ElBaiomy, Jilan Mohamed Youssef

**Affiliations:** ^1^Department of Oral Medicine, Periodontology, Diagnosis and Oral Radiology, Faculty of Dentistry, Mansoura University, Mansoura, Egypt; ^2^Department of Pharmacognosy, Faculty of Pharmacy, Mansoura University, Mansoura, Egypt; ^3^Department of Clinical Pathology, Faculty of Medicine, Mansoura University, Mansoura, Egypt

**Keywords:** Lipocalin-2, nanofibers, local delivery, NGAL, periodontitis

## Abstract

**Background.** The aim of this study was the clinical and laboratory evaluation of using polycaprolactone (PCL) nanofibers loaded with oxytetracycline hydrochloride (OTC) and zinc oxide (ZnO) as an adjunct to mechanical therapy in managing stage II grade A periodontitis patients concerning GCF lipocalin2- levels.

**Methods.** Fifty stage II grade A periodontitis patients (27 women and 23 men, with an age range of 30‒60) were enrolled in the study. The patients were randomly assigned to five equal groups and treated with scaling and root planing (SRP), followed by local application of PCL nanofibers: group I: SRP + PCL loaded with OTC and ZnO, group II: SRP + PCL loaded with OTC, group III: SRP + PCL loaded with ZnO, group IV: SRP + unloaded PCL, and group V: SRP alone. Additionally, 10 healthy subjects with healthy periodontium (group VI) (age- and gender-matched) served as the negative control. Nanofibers were applied in the selected pockets of periodontitis patients in groups I to IV once weekly for two months. All the participants were examined clinically by assessing periodontal indices (gingival index, plaque index, pocket depth, and clinical attachment level), and biochemically by assessing GCF lipocalin-2 levels.

**Results.** Compared to controls, periodontitis groups (I, II, III, IV, and V) showed significant elevation of both clinical parameters and GCF lipocalin2- levels at baseline. In addition, these parameters improved significantly after treatment, which was more pronounced in groups I, II-, and III) compared to groups IV and V. However, it did not reach normal values.

**Conclusion.** In association with SRP, PCL nanofibers loaded with OTC and ZnO had beneficial therapeutic effects at both clinical and laboratory levels.

## Introduction

 Periodontitis is a microbe-driven chronic inflammatory disease. It is primarily characterized by an imbalance between the commensal microbiota and the host response that activates the host’s immune response, causing the destruction of the tooth-supporting tissues, forming periodontal pockets, and eventually leading to tooth loss.^1^ It is one of the most prevalent oral diseases, affecting 20‒60% of the world population.^2^

 The first-line strategy for periodontal disease treatment involves non-surgical periodontal therapy (NSPT), i.e., scaling and root planing (SRP). Although SRP is considered the gold standard in the treatment of periodontitis, it may not remove all the pathogens from the base of deep periodontal pockets because of the complex anatomy of the root and the location of lesions. Therefore, adjunctive therapies have been proposed to improve the NSPT outcomes,^3^ such as antibiotics, antiseptics,^4^ anti-inflammatory drugs,^5^ non-pharmacological agents, and herbal products.^6^

 Systemic use of antibiotics as an adjunct to NSPT has shown positive effects on periodontitis therapy outcomes.^7^ Dysbacteriosis and poor biodistribution are among the adverse effects of systemic drug administration. Toxicity, drug resistance, and gastrointestinal intolerance could result from a high systemic drug dose while trying to achieve and maintain an effective specific site concentration.^8^ Thus, using systemic antibiotics as an adjunct to SRP has become the standard of care only in aggressive and non-responsive forms of periodontitis.^9^

 Thus, to overcome these limitations and improve the treatment of periodontitis outcomes, it is necessary to use local drug delivery systems (LDDs) as they provide more advantages.^8^ These LDDs advantages, compared with systemic ones, include avoidance of gastrointestinal issues and first-pass metabolism by direct drug application at a specific site. Also, achieving more patient compliance by using a controlled release system promotes better results with fewer side effects and reduces the dose frequency.^10^ Generally, LDDs are available in many forms as irrigating systems, fibers, strips, films, injectable gels, and micro/nanoparticle systems.^11^ Nanoparticles are designed to penetrate regions that cannot be reached by other drug delivery systems.^12^

 Nanofibers are among the applied LDDs. Many useful nanofiber fabrication methods are available, such as electrospinning, molecular self-assembly, thermally induced phase separation, interfacial polymerization, and freeze-drying.^13^ Local delivery using nanofibers shares other LDDs characteristics, allowing site-specificity, which leads to a lower overall drug dosage with lower side effects. Furthermore, nanofibers are highly advantageous than others because of their high surface area-to-volume ratios, high porosity, and 3D open porous structures.^14^

 Polycaprolactone’s (PCL) biocompatibility has been approved by theFood and Drug Administration (FDA).^15^ The PCL polymer solution could be fabricated to create an electrospun PCL nanofiber membrane using electrospinning and freeze-drying.^16^ However, PCL is more applicable for long-term than short-term drug delivery systems due to its slow biodegradation rate.^15^

 Different antibiotics, such as oxytetracycline hydrochloride, have been loaded onto PCL nanofibers, where oxytetracycline hydrochloride (OTC) is a bacteriostatic antibiotic that interferes with bacterial protein synthesis and inhibits tissue collagenase activity. Other materials, such aszinc oxide (ZnO), have been loaded onto PCL nanofibers, which have exhibited strong antimicrobial (bacteriostatic) activity even when administered in small amounts.^17,18^

 A biomarker is an objective measure that has been evaluated and confirmed either as an indicator of physiologic health or pharmacologic response to a therapeutic intervention.^19^ These biomarkers can be found in many biologic fluids. Biomarkers in serum can potentially provide information at the patient level, while those in gingival crevicular fluid (GCF) can potentially provide information at the site level. However, saliva contains both local and systemically derived markers and provides information at the patient level.^20^

 Lipocalin-2, mainly released from granules of activated neutrophils,^21^ can bind to iron, fatty acids, prostaglandins, steroids, and matrix metalloproteinases.^22^ It plays a vital role in mediating innate immune responses to bacterial infections and chemoattraction of neutrophils, promoting their maturation, adhesion, extravasation, and phagocyte capacity. In addition, it activates regulatory T cells.^23^ Studies have detected lipocalin-2 in GCF and saliva,^24,25^ suggesting that neutrophil extravasation is the main source of this protein in these biological fluids and gingiva.^25^

 Therefore, this study evaluated the effects of local PCL nanofibers loaded with oxytetracycline hydrochloride and zinc oxide as an adjunct to SRP in treating stage II periodontitis on GCF lipocalin-2 levels.

## Methods

###  Patient selection

 The present study was carried out on 60 subjects of both genders (30 males and 30 females), aged 30 to 60. Fifty patients were diagnosed with stage II grade A periodontitis according to the classification of the American Academy of Periodontology 2017. They were selected from the Department of Oral Medicine and Periodontology Clinic, Faculty of Dentistry, Mansoura University. The study protocol was reviewed and approved by the Ethics Committee of the Faculty of Dentistry, Mansoura University, under the code A1010720. The patients were asked to sign an informed consent after explaining the steps, method, benefits, and potential risks of the treatment to be included in the study. Furthermore, they were informed that they could withdraw at any time without losing any benefits. Another 10 apparently healthy subjects with healthy periodontium were enrolled in the study to determine lipocalin-2 normal levels in GCF and were considered as a negative control group.

###  Inclusion and exclusion criteria 

 The inclusion criteria were stage II grade A periodontitis patients aged 30‒60, with clinical attachment loss (CAL) measuring 3‒4 mm, probing depth >4 mm, and no history of antibiotic or periodontal therapy in the last three months. The exclusion criteria were systemic diseases/conditions that could influence the progression of periodontitis or the treatment response (e.g., diabetes mellitus, smoking, pregnancy).^26,27^

 Those with unacceptable oral hygiene levels during a re-evaluation of phase I therapy, gingival recession, and endodontic involvement, were also excluded.

###  Study design

 Complete medical and dental histories and periodontal charting were obtained from all the patients. Fifty periodontitis patients were randomly assigned to five equal groups (10 patients each): group I: treatment by SRP, followed by local PCL nanofibers loaded with oxytetracycline hydrochloride and zinc oxide; group II: treatment by SRP, followed by local PCL nanofibers loaded with oxytetracycline hydrochloride; group III: treatment by SRP, followed by local PCL nanofibers loaded with zinc oxide; group IV: treatment by SRP followed by local unloaded PCL nanofibers; group V: treatment by SRP alone, as a positive control group; group VI: 10 clinically healthy individuals with healthy periodontium, as a negative control group.

###  Treatment phase

 GCF samples were collected from the selected pockets using sterile paper points. The paper points were inserted into the periodontal pocket (gingival crevice) until light resistance was felt and kept on hold for 30 s. Any paper points contaminated with blood were excluded and discarded. The paper points were placed in sterile Eppendorf tubes containing 100 µL of phosphate-buffered saline solution (pH=7.4) and stored immediately at -80°C for further analysis.

 Gingival crevicular fluid (GCF) samples were collected, and periodontal indices were recorded for all the patients before and eight weeks after starting treatment, while those of the healthy individuals were taken once only. All periodontitis patients underwent phase I therapy, including full-mouth SRP twice in the first week using ultrasonic and hand instruments under local anesthesia if needed, combined with oral hygiene instructions. No antibiotics (either local or systemic) were prescribed during the treatment. In addition, PCL nanofibers were applied in the selected pockets for all the patients in groups (I, II, III, and IV) once weekly for eight weeks as follows:

 Nanofibers were inserted into the selected pockets after careful isolation by cotton rolls and drying of the sites. The fibers were inserted gently from the gingival margins to the base of the pocket to fill the entire pocket without inducing any bleeding. The patients were instructed to avoid any food that might need aggressive chewing and avoid aggressive brushing at selected sites for one week. The patients were instructed to brush the adjacent teeth in the fiber area with great care. In addition, patients were asked to record notes about their experience after the fiber application if there was any adverse effect or abnormal/unusual symptoms throughout the study period.

###  Materials 

 Polycaprolactone (PCL) with a mean molecular weight of 45,000 g/mol, 95% oxytetracycline hydrochloride, and Zn acetate dehydrate were purchased from Sigma-Aldrich (USA). Analytical grades of 99% methylene chloride and 95% methanol were purchased from Fischer Scientific (USA).

###  Methods

 PCL nanofibers were prepared by the freeze-drying technique.^28^

**Figure 1:**
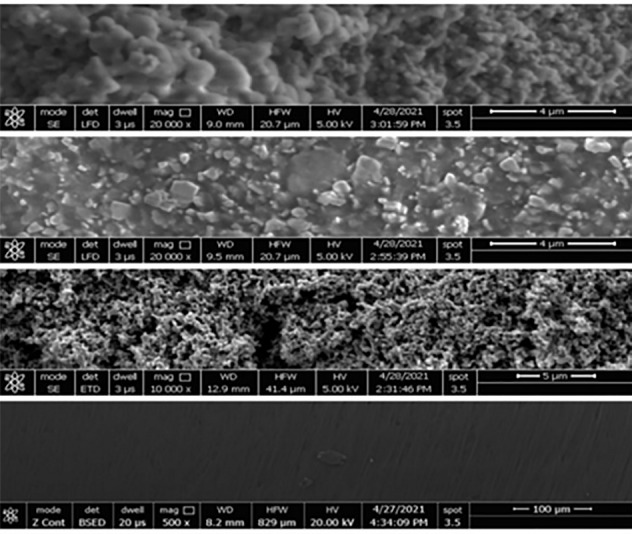
Electron microscope pictures of nanofibers, (A) PCL nanofibers of group I, (B) PCL nanofibers of group II, (C) PCL nanofibers of group III, and (D) PCL nanofibers of group IV.

 Then, they were loaded with the tested antimicrobial in April 2021 (Liver Research Lab-FAB-Lab, Pharmacognosy Department, Faculty of Pharmacy, and Mansoura University, Egypt). Finally, they were tested before clinical use by EM in the National Research Center (Figure 1).

###  Clinical and NGAL assessment

 At baseline and after eight weeks of therapy, the plaque index (PI), gingival index (GI), probing depth (PD), and clinical attachment level (CAL) were measured.GCF lipocalin-2 (NGAL) evaluations were carried out by enzyme-linked immunosorbent assay (ELISA) supplied by Bioassay Technology Laboratory (China) Cat. No. E1719Hu.

###  Statistical analysis

 The data were analyzed using SPSS 22.0 (Armonk, NY: IBM Corp.). Qualitative data were described using numbers and percentages. Quantitative data were described using means and standard deviations for parametric data after testing normality using the Shapiro-Wilk test. Statistical significance was set at P<0.05.

## Results

###  Demographic data

 There were no statistically significant differences between the study and control groups regarding age and gender (Table 1).

**Table 1:** Age and sex of studied groups

**Age and sex**	**Group I ** **N=10 **	**Group II ** **N=10 **	**Group III ** **N=10 **	**Group IV ** **N=10 **	**Group V ** **N=10 **	**Group VI ** **N=10 **	**Test of significance **
Age/years Mean ± SD	49.30±8.22	48.10±9.70	48.30±7.92	46.10±8.33	52.90±7.87	48.20±9.85	F=0.675 P=0.645
Sex Male Female	6(60%) 4(40%)	4(40%) 6(60%)	5(50%) 5(50%)	3(30%) 7(70.0%)	5(50%) 5(50%)	7(70%) 3(30%)	MC P=0.549

MC: Monte Carlo test, F: One-way ANOVA, p: probability

###  Clinical results 

**Plaque index:** There were no significant differences between periodontitis groups (I, II, III, IV, and V) at baseline and after treatment. Meanwhile, there was a significant decrease in PI mean values in groups I, II, III, IV, and V compared with their pre-treatment values; however, they still did not reach the mean of healthy volunteers (group VI) (Tables 2 and 3).

**Table 2:** Comparison of clinical indices between studied groups before treatment

**Before **	**Group I ** **PCL+OTC+ZNO ** **N=10 **	**Group II ** **PCL+OTC ** **N=10 **	**Group III ** **PCL+ZNO ** **N=10 **	**Group IV ** **PCL ** **N=10 **	**Group V ** **SRP ** **N=10 **	**Group VI ** **Healthy sub ** **N=10 **	**Test of significance **
**PI **	2.34±0.28^C^	2.49±0.19^C^	2.33±0.21^C^	2.41±0.30^C^	2.45±0.09^C^	0.16±0.06	F=188.39 P<0.001*
**GI **	1.84±0.17^C^	1.82±0.18^C^	1.79±0.22^C^	1.86±0.309^C^	1.75±0.159^C^	0.19±0.01	F=113.90 P<0.001*
**PPD **	3.30±0.27^C^	3.25±0.34^C^	3.27±0.37^C^	3.39±0.348^C^	3.11±0.38^C^	1.4±0.017	F=54.67 P<0.001*
**CAL **	2.80±0.236^C^	2.57±0.33^C^	2.78±0.31^C^	2.72±0.302^C^	2.62±0.289^C^	0.0±0.0	F=165.85 P<0.001*

F: One-way ANOVA, Significant difference between groups by post hoc Tukey test presented by similar superscripted letters within the same row, *statistically significant. Abbreviations: PI, plaque index; GI, gingival index; PD, probing depth; CAL, clinical attachment loss

**Table 3:** Comparison of clinical indices between studied groups after treatment

**After **	**Group I ** **PCL+OTC+ZNO ** **N=10 **	**Group II ** **PCL+OTC ** **N=10 **	**Group III ** **PCL+ZNO ** **N=10 **	**Group IV ** **PCL ** **N=10 **	**Group V ** **SRP ** **N=10 **	**Group VI ** **healthy ** **N=10 **	**Test of significance **
**PI **	0.809±0.085^F^	0.850±0.08^F^	0.821±0.081^F^	0.872±0.064^F^	0.865±0.055^F^	0.167±0.07^a^	F=129.53 P<0.001*
**GI **	0.331±0.049^DE^	0.341±0.058^DE^	0.353±0.072^DE^	0.774±0.195^F^	0.434±0.129^F^	0.191±0.06^DE^	F=15.45 P<0.001*
**PPD **	1.565±0.17^DE^	1.60±0.175^DE^	1.67±0.244^DE^	2.82±0.352^F^	2.19±0.35^F^	1.464±0.02^DE^	F=44.88 P<0.001*
**CAL **	1.31±0.137^FDE^	1.47±0.24^FDE^	1.53±0.16^FDE^	2.149±0.33^F^	2.03±0.36^F^	0.0±0.0	F=101.95 P<0.001*

F: One-way ANOVA; similar superscripted letters within the same row denote significant difference between groups by post hoc Tukey test, *statistically significant. A: significant with group I, B: significant with group II, C: significant with group III, D: significant with group IV, E: significant with group V, F: significant with group V

**Gingival index:** Before treatment, there were significant differences in GI mean values between group VI (negative control) and all the periodontitis groups (I, II, III, IV, and V). However, there were no significant differences between periodontitis groups (I, II, III, IV, and V). After treatment, all the LDD-treated groups ([I, II, and III] using PCL loaded with drugs) showed improvements in GI. The lowest mean GI was detected in group I, followed by groups II and III, but they did not reach the mean value of healthy participants (group VI). A comparison of groups I, II, and III revealed no significant differences in mean GI. In addition, there was no significant difference when they were compared with group VI. However, there was a significant decrease in groups I, II, and III compared with groups IV and V, with no significant difference between groups IV and V (Tables 2 and 3).

**Pocket depth: **At baseline, there were significant differences between the healthy group (VI) and all the periodontitis groups, while there were no significant differences between all the periodontitis groups (I, II, III, IV, and V). Although PD decreased significantly after treatment, and the lowest mean PD was detected in group I, followed by groups II and III, they did not reach the mean value of healthy participants of group VI. All the periodontitis patients in groups I, II, and III demonstrated no significant difference in mean PD compared to each other. There was a significant decrease between groups I, II, and III (groups treated with PCL loaded with drugs) compared with groups IV and V (groups treated with SRP), with no significant difference between groups IV and V (Tables 2 and 3).

**Clinical attachment level:** Before treatment, there were significant differences between the healthy group (VI) and all the periodontitis groups, with no significant differences between the periodontitis groups (I, II, III, IV, and V). After treatment, the lowest mean CAL was detected in group I, followed by groups II and III. Groups I, II, and III demonstrated no significant differences in mean CAL compared to each other. Also, there were no significant differences between groups IV and V. However, there were significant decreases in groups I, II, and III compared to groups IV and V (Tables 2 and 3).

**Biochemical results: **GCF lipocalin-2 pre-treatment mean value was significantly different between the healthy group (VI) and the periodontitis groups. After treatment, its level in all the periodontitis groups decreased significantly compared to baseline; the lowest level of lipocalin-2 was detected in group I, followed by groups II and III. Groups I, II, and III showed significant differences compared with groups IV and V. In addition, groups IV and V showed a statistically significant difference compared to group VI. However, there were no significant differences between groups I, II, and III compared with group VI (Table 4).

**Table 4:** Comparison of lipocalin-2 levels between studied groups before and after treatment

**Lipocalin-2 **	**Group I ** **PCL+OTC+ZNO ** **N=10 **	**Group II ** **PCL+OTC ** **N=10 **	**Group III ** **PCL+ZNO ** **N=10 **	**Group IV ** **PCL ** **N=10 **	**Group V ** **SRP ** **N=10 **	**Group VI ** **Healthy ** **N=10 **	**Test of significance **
**Before **	247.50±22.1^F^	252.4±20.36^F^	226±47.39^F^	240.10±37.4^F^	233.6±31.2^F^	187.30±14.8	F=7.001 P<0.001*
**After **	180.90±23.87^DE^	183.20±6.08^DE^	185.60±12.42^DE^	219.30±40.30^F^	220.7±12.67^F^	187.30±14.8	F=3.35 P=0.017*

**Figure 2:**
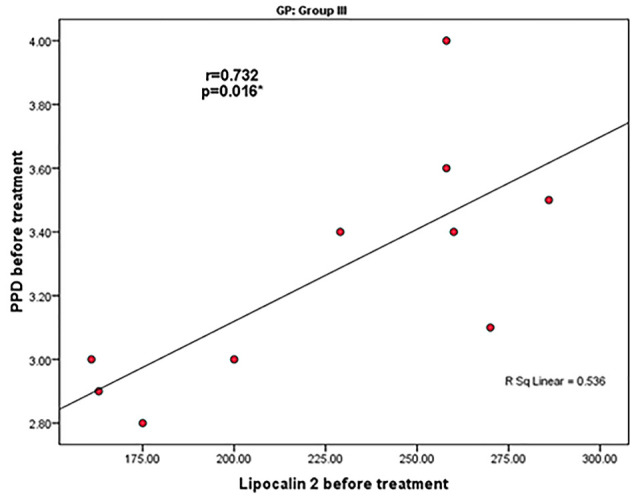
Before treatment, a statistically significant positive correlation was detected between lipocalin-2 and PPD in group III (r=0.732) without any other significant correlation.

**Figure 3:**
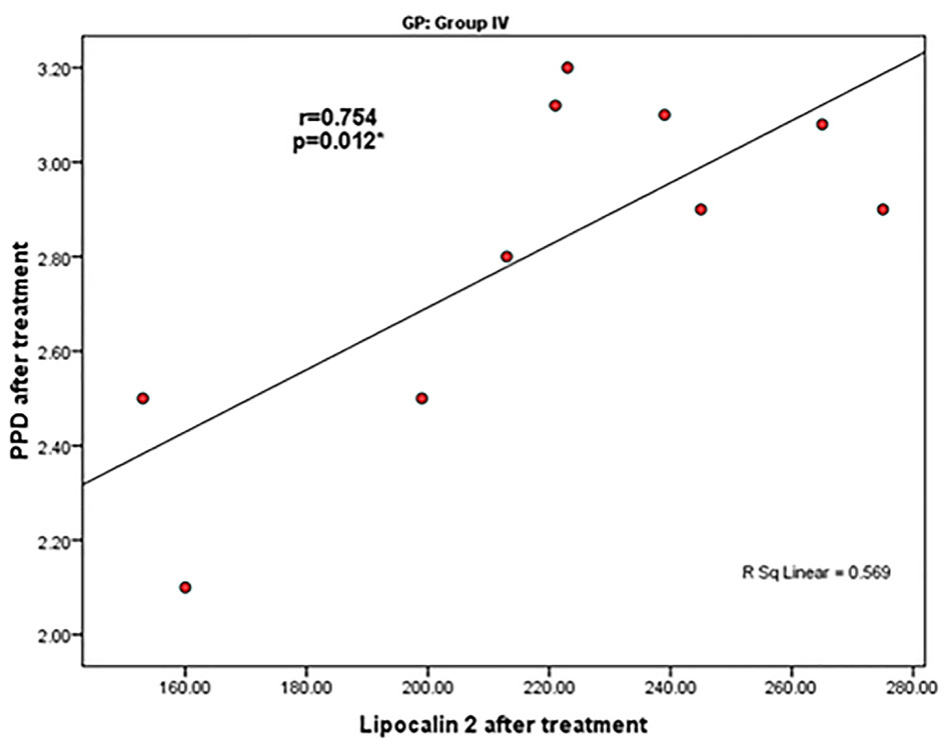
After treatment, there was a statistically significant positive correlation between lipocalin-2 and PPD in group IV (r=0.754).

F: One-way ANOVA; similar superscripted letters within the same row denote significant difference between group VI and all studied groups by post hoc Tukey test. *statistically significant A: significant with group I, B: significant with group II, C: significant with group III, D: significant with group IV, E: significant with group V, F: significant with group VI,

 Before treatment, there was a statistically significant positive correlation between lipocalin-2 levels and PPD in group III (r=0.732) without any other significant correlations (Figure 2). After treatment, there was a statistically significant positive correlation between lipocalin-2 and PPD in group IV (r=0.754) (Figure 3).

## Discussion

 Adjunctive pharmacotherapy for periodontitis management becomes mandatory in some cases. Local drug delivery systems (LDDs) are one of such commonly used practices. LDDs provide curative effects by carrying either antimicrobial, inflammation modulatory, or bone tissue regeneration active agents.^29^ Nanoscale LDDs, such as nanofibers, are biocompatible and biodegradable polymers, with thermal stability and good mechanical properties, in addition to high efficiency in drug loading as a result of their large surface area and small size that allow their penetration to regions that may be inaccessible to other delivery systems.^30^ Therefore, nanofibers have been suggested as promising adjuncts to SRP for the efficient treatment of periodontitis. Moreover, the combined incorporation of OTC and ZnO into PCL nanofibers could provide a more sustained release of antimicrobial drugs^30^ for more effectiveness. Therefore, the present study investigated the effects of adjunctive PCL nanofibers on clinical status and GCF lipocalin-2 levels in periodontitis patients.

 Baseline clinical parameters (PI,GI, PD, and CAL) and GCF lipocalin-2 levels showed no significant differences between the periodontitis groups. However, there were significant improvements in all the clinical parameters in all the periodontitis groups compared to baseline after treatment. The present study showed that improvements in clinical parameters were more evident in LDD-treated groups (I, II, and III) compared to groups IV and V. The lower improvements in SRP-treated groups could be attributed to the limited effect of SRP in deep periodontal pockets, complex structure, and inaccessible areas; hence the persistence of periodontal pathogens and persistent inflammation.^31^ This finding confirms the beneficial use of adjunctive therapy in addition to SRP for potentiating the SRP effect tocompletely eradicate the pathogens and eliminate the associated inflammation.

 Regarding group II, there were significant improvements in clinical and biochemical parameters compared to baseline values. This may be attributed to the oxytetracycline hydrochloride use that provides anti-collagenase activity, preventing collagen destruction and inhibiting alveolar bone resorption, in addition to its antibacterial effect. These findings were supported by Chandrashekar et al.,^32^who reported significant reductions in clinical parameters (PI,GI, PD, and CAL) and aspartate transaminase levels in patients using oxytetracycline hydrochloride compared to SRP alone. Similarly, Chaturvedi et al.^33^ reported a similar effect of PCL nanofibers loaded with doxycycline as adjunctive therapy to SRP in the chronic periodontitis patients’ pockets treatment.^34^

 Regarding group III,there were significant improvements in clinical and biochemical parameters compared to baseline values. This may be attributed to the biocompatibility, biosafety, and nontoxic metal oxide nanoparticles (ZnO NPs) that act as potent antibacterial agents against a broad range of bacteria (Gram-positive and Gram-negative) and fungi. These findings were supported by Münchow et al.,^35^ who successfully incorporated ZnO NPs into PCL-based electrospun membranes and improved the bioactivity of the membranes for GTR/GBR applications, leading to enhanced periodontal regeneration.These findings were also supported bySeo et al.^36^** w**ho demonstrated that the PCL membranes carrying ZnO nanoparticles inhibited bacterial adhesion without affecting the viability of osteoblasts, suggesting the potential application of ZnO in GTR to increase the antibacterial activity of membranes.

 Concerning group I, there were significant improvements in clinical and biochemical parameters compared to baseline values. This may be attributed to the combined antibacterial activity provided by oxytetracycline hydrochloride and zinc oxide, illustrating a slightly greater improvement in group I compared to groups II and III. Our findings were supported by Dias et al.,^30^who studied incorporating two antibacterial agents, OTC and ZnO, to PCL nanofibers to treat periodontal diseases. They found that PCL nanofibers loaded with OTC and PCL loaded with OTC–ZnO displayed good antibacterial activity against a mixed bacterial culture, and PCL–OTC/ZnO nanofibers showed considerable potential as a drug delivery system to treat periodontal diseases.

 Unlike the periodontitis groups (I, II, and III) that were treated by SRP followed by PCL nanofibers loaded with antimicrobial agents, groups IV and V were treated by SRP + unloaded PCL and SRP alone, respectively. The absence of the active antimicrobial agent in group IV (to exclude the possibility of PCL’s role in bacteria and/or tissue healing) resulted in a similar final treatment for periodontitis patients in both groups (IV and V). However, both groups showed clinical and biochemical improvements that could be attributed to the successful role of SRP and patient oral hygiene in decreasing bacterial burden and inflammation.^31^

 Baseline GCF lipocalin-2 levels in the current study were not significantly different between all the periodontitis groups (I, II, III, IV, and V). However, its level in these groups was significantly higher compared to the healthy group (VI).

 Consistent with this finding, Tan et al.^37^demonstrated elevated levels of serum and salivary lipocalin-2 in periodontitis patient that was correlated with disease severity. In this respect,Westerlund et al.^25^ reported higher lipocalin-2 activity in the GCF of periodontitis patients, compared to periodontally healthy controls, and demonstrated immunohistochemically that extravasated PMNs were its major source. Supporting this issue, lipocalin-2 deficiency may decrease neutrophil infiltration, myeloperoxidase activity, and expression of TNF-α and IL-1β.

 Eight weeks after starting therapy, the levels of GCF lipocalin-2 decreased significantly in LDD-treated periodontitis groups (I, II, and III), becoming non-significantly different from the clinically healthy group (VI). However, in groups IV and V, the GCF lipocalin-2 levels decreased significantly but did not reach normal levels as they showed a significant difference from the clinically healthy group. This finding can be explained by the beneficial effect of LDD used in groups I, II, and III, indicating the potentiation of the SRP effect and leading to the amelioration of inflammation and rapid recovery.

 Lipocalin-2 was found to be correlated with PD before and after treatment in the periodontitis group IV, while it was correlated with CAL in the periodontitis group V after treatment in the present study (Figures 2 and 3). This finding partially agreed with the previous reports of Tan et al.,^37^ who found significant and strong correlations between lipocalin-2 and clinical parameters. On the other hand, Ceylan et al.^38^ demonstrated that lipocalin-2 and TNF-α levels significantly and positively correlated with periodontal clinical parameters, and its level significantly changed in response to non-surgical periodontal therapy in periodontitis patients. The difference between the present study and previous findings may be attributed to different sample sizes.

## Conclusion

 Combined incorporation of OTC and ZnO into PCL nanofibers could be a potential curative tool in chronic periodontitis as an adjunct to SRP as it improved the clinical parameters and reduced the GCF lipocalin-2 levels.

## Acknowledgments

 This work was undertaken in Mansoura University, Faculty of Medicine, Department of Clinical Pathology, and in the Faculty of Dentistry, Department of Periodontology and Oral Medicine clinics.

## Competing Interests

 The authors declare that they have no competing interests.

## Authors’ Contributions

 JMY: designed the study, controlled all the study procedures, analyzed the clinical data, and wrote and edited the manuscript. AAB: performed the biochemical assessment and analyzed the data. FAB: controlled all the steps of preparation and loading of the PCL nanofibers. MFK: obtained the biological samples and clinical data. Each author contributed to the final document of the manuscript and discussed the finding.

## Funding

 There was no financial support for the study.

## Availability of data

 The data that support our results are available if requested from the corresponding author, JMY. The data are not available to the public, as they contain private information about research volunteers.

## Ethics Approval

 The study methodology was authorized by the Mansoura Faculty of Dentistry’s Ethics Committee under the codr A10010720.
